# Melatonin Modulates Dendrite Maturation and Complexity in the Dorsal- and Ventral- Dentate Gyrus Concomitantly with Its Antidepressant-Like Effect in Male Balb/C Mice

**DOI:** 10.3390/ijms21051724

**Published:** 2020-03-03

**Authors:** Gerardo Bernabé Ramírez-Rodríguez, Diana Montserrat Palacios-Cabriales, Leonardo Ortiz-López, Erika Montserrat Estrada-Camarena, Nelly Maritza Vega-Rivera

**Affiliations:** 1 Laboratory of Neurogenesis, Division of Clinical Investigations, National Institute of Psychiatry “Ramón de la Fuente Muñiz”, Calzada Mexico-Xochimilco No. 101, Mexico City C.P. 14370, Mexico; diana.cabrico@gmail.com (D.M.P.-C.); leosan@imp.edu.mx (L.O.-L.); 2 Laboratory of Neuropsychopharmacology, Division of Neurosciences, National Institute of Psychiatry “Ramón de la Fuente Muñiz”, Calzada Mexico-Xochimilco No. 101, Mexico City C.P. 14370, Mexico; estrada@imp.edu.mx

**Keywords:** adult neurogenesis, melatonin, dorsal-ventral hippocampus, doublecortin, forced-swim, depression

## Abstract

Adult neurogenesis occurs in the dentate gyrus (DG) of the hippocampus. New neurons help to counteract the effects of stress and several interventions including antidepressant drugs, environmental modifications and internal factors act pro-neurogenic with consequences in the dorsal and ventral DG. Melatonin, the main product synthesized by the pineal gland, induces antidepressant-like effects and modulates several events of the neurogenic process. However, the information related to the capability of melatonin to modulate dendrite maturation and complexity in the dorsal and ventral regions of the DG and their correlation with its antidepressant-like effect is absent. Thus, in this study, we analyzed the impact of melatonin (0, 0.5, 1, 2.5, 5 or 10 mg/kg) administered daily for fourteen days on the number, dendrite complexity and distribution of doublecortin (DCX)-cells in the dorsal-ventral regions of the DG in male Balb/C mice. Doublecortin is a microtubule-associated protein that is expressed during the course of dendritic maturation of newborn neurons. Also, we analyzed the impact of melatonin on despair-like behavior in the forced swim test. We first found a significant increase in the number and higher dendrite complexity, mainly with the doses of 2.5, 5 and 10 mg/kg of melatonin (81%, 122%, 78%). These cells showed more complex dendritic trees in the ventral- and the dorsal- DG. Concomitantly, the doses of 5 and 10 mg/kg of melatonin decreased depressant-like behavior (76%, 82%). Finally, the data corroborate the antidepressant-like effect of melatonin and the increasing number of doublecortin-associated cells. Besides, the data indicate that melatonin favors the number and dendrite complexity of DCX-cells in the dorsal- and ventral- region of the DG, which may explain part of the antidepressant-like effect of melatonin.

## 1. Introduction

Hippocampal neurogenesis occurs in the DG of mammals, including humans [1]. The neurogenic process is affected by animal models of stress [2,3]. Also, the increased generation of new neurons helps to buffer the effects of stress in rodents [2,3]. Interestingly, some events of this process are altered in humans diagnosed with depression [4,5].

Depression is a multifactorial neuropsychiatric disorder affecting a high proportion of human around the world [6,7]. Thus, several attempts have been established to counteract depression being the pharmacological therapy the first line of treatment. Antidepressant drugs have shown their pro-neurogenic potential and their capability to revert depressive behavior [8,9,10,11,12]. However, in recent years, it has been proposed that some molecules or environmental interventions, such as physical activity, cognitive enrichment or internal factors may act as an adjuvant for treatment of depression [13,14].

In this regard, melatonin, the main product synthesized by the pineal gland, exhibits the capability to act as a regulator of the generation of new neurons in the DG of the hippocampus in rodents and produces antidepressant-like effects [15,16,17,18,19,20]. Interestingly, melatonin alterartions occur in patients diagnosed with depression which show alteration in the circadian rhythms [21,22,23].

Besides, previous studies showed consistent results of melatonin on several events of the neurogenic process in C57Bl/6 mice or CD1 mice (i.e., [15,16,17,24]). Explicitly, melatonin favored cell proliferation, survival, intermediate stages of adult hippocampal neurogenesis during ageing of Balb/C mice [25] and the structural plasticity of mossy fibers in granule cells [26]. Also, melatonin stimulates dendrite maturation of doublecortin (DCX)-associated cells in C57Bl/6 mice [24]. DCX is a microtubule-associated protein involved in cell migration and neuritogenesis [27]. Additionally, DCX is expressed on the course of dendritic maturation of newborn neurons [28,29]. Moreover, DCX localizes with the dendritic cytoplasm [29,30].

Interestingly, the pro-neurogenic or anti-depressive like effects of melatonin analyzed in separated studies after the administration of different doses of melatonin ranging from 2.5 to 10 mg/kg, i.e., [15,16,17,19,21,22,23,24,25,26,27,28,29,30,31,32,33,34]. However, the information related to the capability of melatonin to modulate dendrite maturation and complexity in the dorsal- and ventral- regions of the DG and their relationship with its antidepressant-like effect is not known. This aspect is relevant, especially because dorsal-DG is mainly involved in learning and memory processes, whereas the ventral-DG participates in the modulation of fear and anxiety [35,36,37,38,39,40]. Also, several antidepressant-drugs and environmental interventions showed uniform or region-specific effects on neurogenesis in both regions of the DG (for review see [41]).

We thus here hypothesized that melatonin could promote differential regulation of the generation of new neurons, here tested with the identification of DCX-associated cells, in the dorsal- and ventral- DG and its antidepressant-like effect following a dose-dependent manner. Thus, our data indicate that melatonin favors the generation of new neurons and their dendrite maturation in both regions, dorsal and ventral, of the DG concomitantly to its antidepressant-like effects in the FST.

## 2. Results

### 2.1. Melatonin Increases the Number of Doublecortin-Associated Cells in the Dentate Gyrus

**Figure 1:**
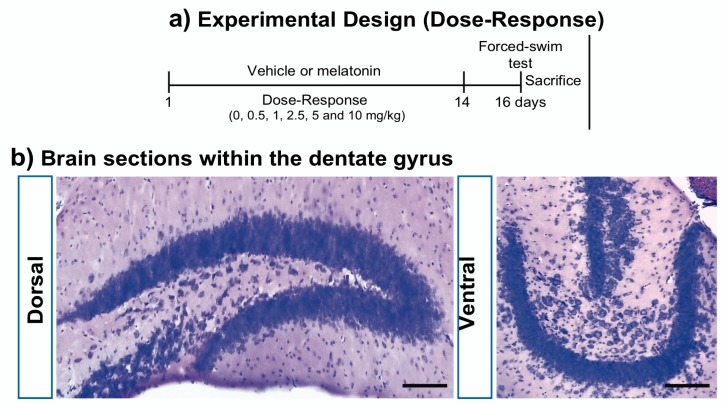
Administration of melatonin. (**a**) Male Balb/C male mice were kept under normal housing conditions until the age of 8 weeks. Mice were treated with vehicle (CTL) or with different doses of melatonin for 14 days. All doses of melatonin were injected at 19:00 hours. The behavioral test was performed 36 hours after the last drug administration (Day 16). *n* = 9–10 per group. Once behavior was assayed, mice were sacrificed to perform doublecortin analysis in both coronal and transversal sections within the dentate gyrus. Representative images after Nissl staining are shown in (**b**). Scale bars represent 100 μm. *n* = 6 per group.

**Figure 2:**
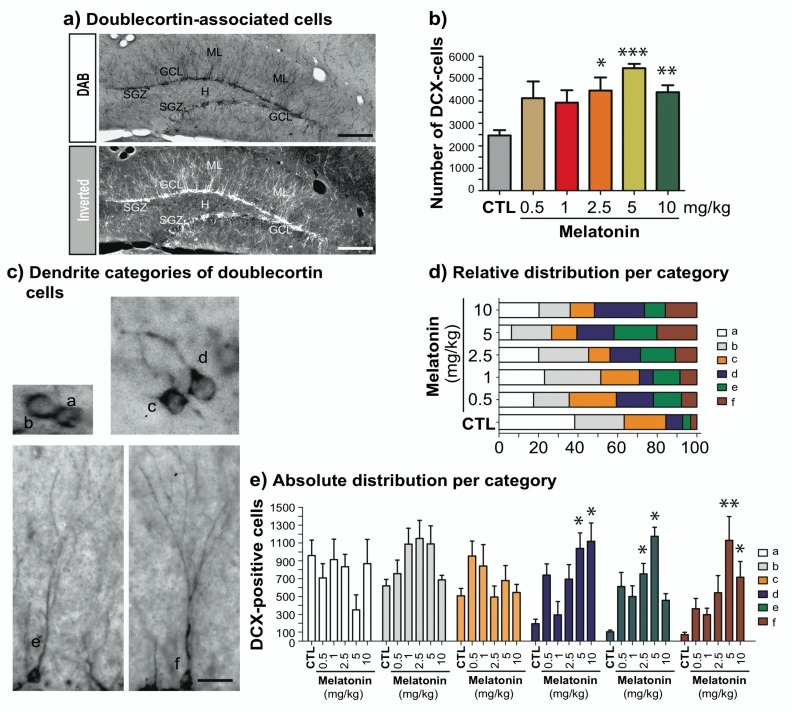
Doublecortin-associated cells located in the dentate gyrus of adult male Balb/C mice. (**a**) Mice were intraperitoneally injected either with 0.5, 1, 2.5, 5 and 10 mg/kg of melatonin or vehicle for 14 days. Coronal sections within the hippocampus are shown, and doublecortin (DCX)-associated cells were identified with an anti-doublecortin antibody. Representative pictures show the subgranular zone delimited by DCX-associated cells (SGZ). Some cells are integrated into the granular cell layer (GCL), and some of their dendrites reach the molecular layer (ML). Images also show the hilus (H). Scale bars represent 200 μm. (**b**) Chronic melatonin (2.5, 5 and 10 mg/kg) administration induced a significant increase in DCX-associated cells in comparison with the vehicle. Error bars represent standard error of the mean (SEM). Asterisks indicate *p* = 0.045, 0.002, 0.039; respectively. Representative bright-field images corresponding to the different morphologies (**a**–**f**) are also shown in panel (**c**). Scale bar represents 20 μm. (**d**) Relative distribution per category of DCX-labelled cells is shown and (**e**) absolute quantification based on a percentage per category and the total number of DCX-labelled cells confirmed a significant increase in more appearing mature neurons caused by higher doses of melatonin. Error bars represent SEM. Asterisks indicate *p* = 0.001 for category “d”, *p* = 0.028, <0.001 for category “e”; and *p* < 0.001, 0.031 for category “f”. *n* = 6 mice per group.

We first determined the effects of the doses of melatonin (0.5, 1, 2.5, 5 or 10 mg/kg; Figure 1) administered for 14 days on DCX-associated cells (Figure 2) located in the subgranular zone of the DG (Figure 2a). The total number of DCX-associated cells in the DG of mice treated with the different doses of melatonin showed significant increase after the administration of treatments ranging from 2.5 to 10 mg/kg of melatonin (81%, 122%, 78%) compared to vehicle (*p* = 0.045, 0.002, 0.039) treated mice (F_5,35_ = 4.12, *p* = 0.006; Figure 2b).

### 2.2. Melatonin Modulates Dendrite Maturation of Doublecortin-Associated Cells in the Dentate Gyrus

We investigated whether the doses administered of melatonin modify dendrite organization of DCX-associated cells in male Balb/C mice (Figure 2c). We categorized DCX-associated cells according to their dendrite morphology (Figure 2d). Two-way ANOVA interaction between factor A (dose) and factor B (category) yielded significant interaction (F_25,215_ = 6.007, *p* < 0.001; Figure 2d). Analysis of the proportion of DCX-associated cells per category indicated that mice treated with melatonin (0.5, 1, 2.5, 5 or 10 mg/kg) showed lower cells of the category “a” than that found in vehicle-treated mice (*p* < 0.001). However, there were no differences in categories “b and c” in comparison to the vehicle group but in category “d” 10 mg/kg of melatonin increased the proportion of this type of cells (*p* < 0.001). Moreover, mice treated with 2.5 and 5 mg/kg of melatonin showed an increased proportion of DCX-cells of category “e” compared to the control group (*p* = 0.016, <0.001; respectively). Interestingly, 5 and 10 mg/kg of melatonin produced an increased proportion of DCX-cells with a more elaborated dendritic tree in comparison to the control mice (*p* = 0.001, 0.026; respectively). However, we did not find differences between 5 and 10 mg/kg of melatonin (*p* = 0.32).

Analysis of absolute numbers per category within the two-way ANOVA showed an interaction between factor A (dose), and factor B (category) yielded significant interaction (F_25,215_ = 3.15, *p* < 0.001; Figure 2e). Absolute numbers per category indicated that melatonin tested at the different doses does not induce substantial changes in the number of cells of categories “a–c”. However, the absolute number of category “d” was increased in mice treated with 5 and 10 mg/kg of melatonin (*p* ≤ 0.001) in comparison to the control group. Also, the absolute number of category “e” increased with 2.5 and 5 mg/kg of melatonin (*p* = 0.028, <0.001; respectively). Finally, the number of cells of category “f” increased with the higher doses of melatonin (5 and 10 mg/kg; *p* < 0.001, 0.031; respectively). Also, we did not find differences between 5 and 10 mg/kg of melatonin (*p* = 0.062) for category “f”. These results show that higher doses of melatonin increase DCX-associated cells with more mature dendrite morphology.

### 2.3. Melatonin Increases the Number of Doublecortin-Associated Cells in the Dorsal Rather Than in the Ventral Dentate Gyrus

**Figure 3:**
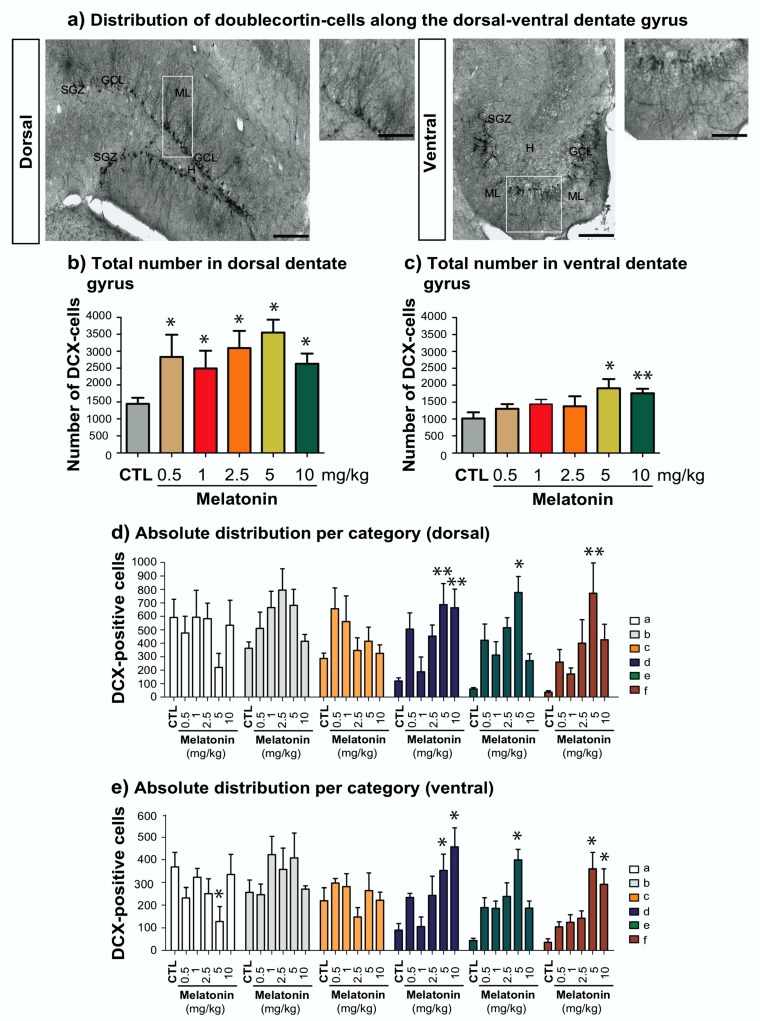
Distribution of doublecortin-cells along the dorsal-ventral dentate gyrus of adult male Balb/C mice. Mice were intraperitoneally injected either with 0.5, 1, 2.5, 5 and 10 mg/kg of melatonin or vehicle for 14 days. Coronal and transversal sections within the dorsal or ventral dentate gyrus are shown in (**a**). Representative pictures show the subgranular zone delimited by DCX-associated cells (SGZ). Some cells are integrated into the granular cell layer (GCL), and some of their dendrites reach the molecular layer (ML). Images also show the hilus (H). Scale bars 50 μm. Scale bars represent 40 and 200 μm. (**b**) Melatonin induced a significant increase in DCX-associated cells in the dorsal-dentate gyrus. Error bars represent SEM. Asterisks indicate *p* < 0.05 in comparison with the vehicle. (**c**) Also, higher doses of melatonin increased the number of DCX-cells in the ventral-dentate gyrus. Error bars represent standard error of the mean SEM. Asterisks indicate *p* = 0.021, 0.008 for 5 and 10 mg/kg. (**d**) Absolute quantification of DCX-associated cells per category cells in the dorsal dentate gyrus showed that the higher doses of melatonin (2.5, 5 and 10 mg/kg) impact cells of categories “d–f”. Error bars represent (SEM). Asterisks indicate *p* = 0.009 for category “d”, *p* < 0.001, <0.001 for category “e”; and *p* < 0.001 for category “f”. *n* = 6 mice per group. (**e**) Absolute quantification of DCX-associated cells per category cells in the ventral dentate gyrus showed that the highest doses of melatonin (5 and 10 mg/kg) impact cells of categories “d–f”. Error bars represent SEM. Asterisks indicate *p* = 0.011, <0.001 for category “d”, *p* < 0.001 for category “e”; and *p* < 0.001, 0.015 for category “f”. *n* = 6 mice per group.

Furthermore, we quantified the number of DCX-associated cells in the dorsal- and ventral DG (Figure 3a–c). The analysis revealed that all of the doses of melatonin increased the number of DCX-associated cells (*p* < 0.05) in comparison to the control group (H = 11.63, d.f. = 5, *p* = 0.040). However, in the ventral-DG (F_5,35_ = 2.52, *p* = 0.049), we found that 5 and 10 mg/kg of melatonin increase the number of DCX-cells (*p* = 0.021, 0.008). These results suggest that higher doses of melatonin have a uniform effect of favoring the increasing number of DCX-associated cells in both regions of the dentate gyrus.

### 2.4. Melatonin Modulates Dendrite Maturation of Doublecortin-Associated Cells in the Dorsal Rather Than in the Ventral Dentate Gyrus

Besides, we analyzed the absolute numbers per category in the dorsal- (Figure 3d) and ventral-(Figure 3e) DG in terms of their dendrite complexity. In the dorsal-DG exists an interaction between factor A (dose) and factor B (category) yielded significant interactions (F_25,215_ = 2.32, *p* < 0.001; Figure 3d). We found that 5 and 10 mg/kg of melatonin increased the number of cells of category "d" in comparison to the control group (*p* = 0.008, 0.009). However, in cells of the category "e and f" the dose of 5 mg/kg of melatonin increased their number (*p* < 0.001; <0.001). Moreover, the analysis in the ventral-DG showed an interaction between factor A (dose) and factor B (category) yielded significant interactions (F_25,215_ = 2.88, *p* < 0.001; Figure 3e). Again, higher doses of melatonin, 5 and 10 mg/kg, increased the number of DCX-cells of categories “d” (*p* = 0.011, <0.001) and “f” (*p* < 0.001, 0.015).

### 2.5. Melatonin Produces Antidepressant-Like Behaviour, and it Correlates with the Dendrite Maturation of DCX-Associated Cells

**Figure 4:**
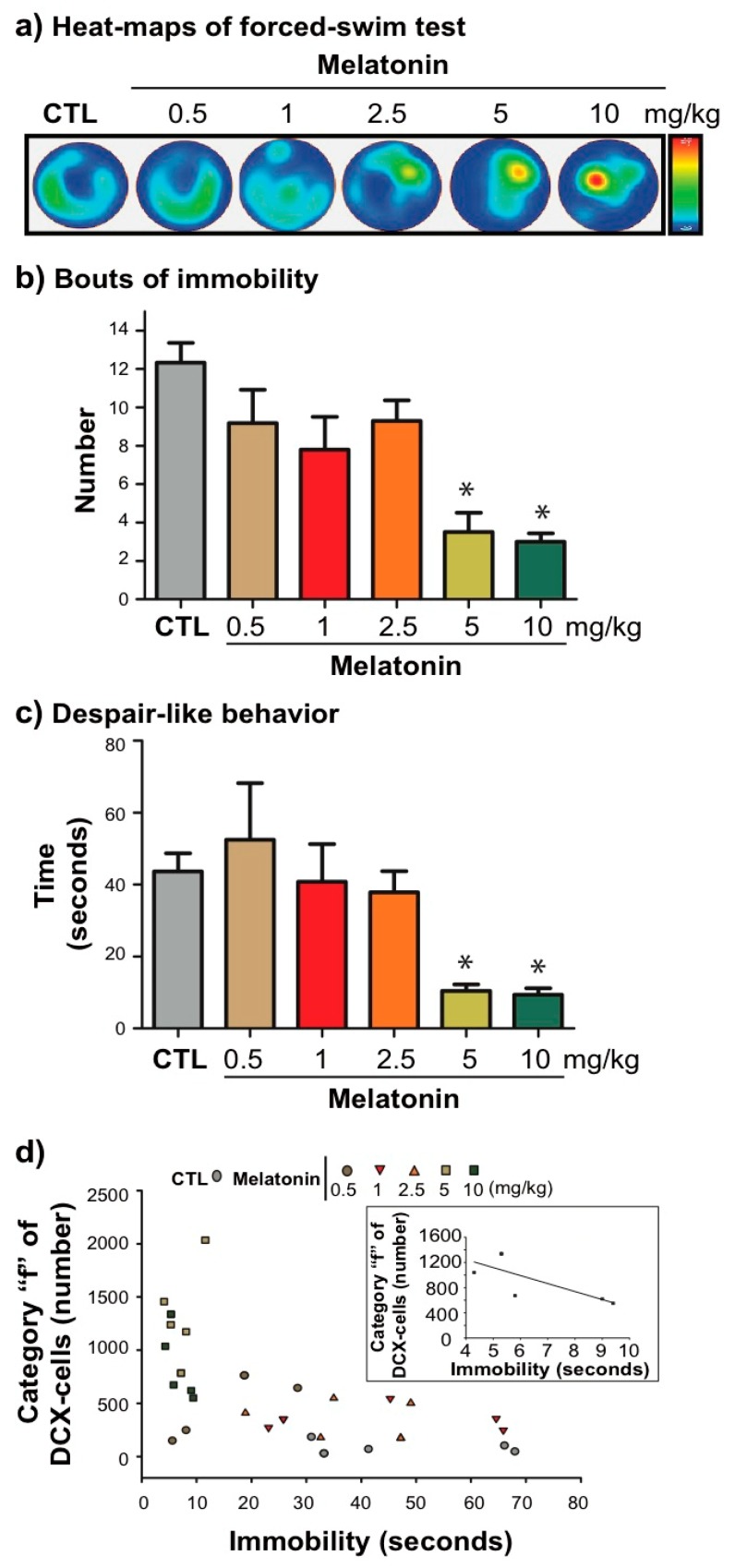
Melatonin decreases the despair behavior in the forced swim test. Male BalbC mice were treated with different doses of melatonin (0.5, 1, 2.5, 5, and 10 mg/kg) for 14 consecutive days before being exposed to the forced swim test. Representative heat maps per group are shown in (**a**). (**b**) Bouts of immobility. Error bars represent standard error of the mean (SEM). Asterisks indicate *p* < 0.001. (**c**) Immobility behavior. Error bars represent SEM. Asterisks indicate *p* < 0.05. *n* = 9–10 mice per group. (**d**) Scatter graph of immobility time and the number of DCX-cells with more mature dendrite complexity (category “f”) in mice treated with the different doses of melatonin. Insert in d corresponds to the Pearson correlation between category “f” of DCX-cells and the immobility behavior in mice treated with 10 mg/kg. *p* = 0.047, Pearson r = −07641. *n* = 6.

Finally, we analyzed the number of immobile episodes and the immobility behavior during FST in male Balb/C mice (Figure 4a). As we found in our previous studies [15,16,17,19,28,29,30,31], the higher doses of melatonin (5 and 10 mg/kg) produced less immobile episodes (*p* < 0.001) and immobility behavior (*p* < 0.05) than the vehicle group (F_5,57_ = 7.77, *p* < 0.001; H = 23.46, d.f. =5, *p* < 0.001; respectively. Figure 4b,c).

Also, we correlate the absolute number of DCX-associated cells corresponding to category "f", with the immobility behavior in the FST (Figure 4d). We found a negative correlation of DCX-cells of category “f” with the decrease immobility behavior in mice treated with 10 mg/kg of melatonin (*p* = 0.047, Pearson r = −0.7641, *n* = 6) but not with 5 mg/kg of melatonin (*p* = 0.36, Pearson r = −0.45, *n* = 6). In addition, we analyzed the influence of the DCX-associated cells of category “f” located in the dorsal- or ventral- DG with the performance of the mice in the FST, but there were no differences. These results suggest the relationship between a higher number of DCX-neurons with more mature dendrite complexity caused by 10 mg/kg of melatonin with the decrease despair behavior in the FST.

## 3. Discussion

In this study, we explored the effects of different doses of melatonin on the number and dendrite morphology of DCX-associated cells in the dorsal- and ventral- DG. We found that higher doses of melatonin increase the total number of DCX-associated cells. Preferentially affected immature neurons with more mature dendrite arborization. These effects occur in both regions of the DG, but the higher number of cells was found in the dorsal-DG. Also, we confirmed the antidepressant-like effect produced with higher doses of melatonin (5 and 10 mg/kg) in male Balb/C mice. Interestingly, the increasing number and dendrite maturation of DCX-associated cells correlate with the decrease despair-like behavior in mice treated with 10 mg/kg of melatonin.

Melatonin is one of the key individual regulators of adult hippocampal neurogenesis, and its effects have been demonstrated at different levels of the neurogenic process including cell proliferation, survival and the generation of new neurons but also on the structural plasticity of axons of granule cells [15,16,17,18,19,20,21,22,23,24,26]

Interestingly, new neurons buffer the effects of stress [2,3] and increase evidence have supported the antidepressant-like effect of melatonin in predictive animal models of stress, i.e., [15,16,17,19,31,32,33,34]. However, the antidepressant-like effect of melatonin is reporting in different studies after the administration of different doses of melatonin ranging from 5 to 10 mg/kg [15,16,17,19,28,29,30]). In the same sense, the pro neurogenic effects of melatonin are reporting in the same range of doses [15,16,17,19,31,32,33,34]. However, to our knowledge, there is no study establishing the range of the doses that may affect hippocampal neurogenesis and its antidepressant-like effect of melatonin. Although to the latter aspect, we previously showed that 5 and 10 mg/kg of melatonin administered for 14 days decrease despair-like behavior in male Balb/C mice tested in the forced-swim paradigm [34].

In the present study, administration of some of the doses of melatonin (2.5, 5 and 10 mg/kg) for 14 days increased the total number of DCX-associated cells in the DG. This finding is in line to the increasing number of DCX-cells found in previous studies performed in female C57Bl6 or Balb/C mice [24,25] or to studies conducted in animal models in which the decrease of immature neurons by irradiation was preventing by melatonin. In those studies, the neurogenesis related marker used was DCX [42,43].

Besides, DCX-protein is associating to microtubules along the dendrite, and it expresses from the initial steps of neuronal differentiation till more advanced stages of differentiation, including dendritic branching [27]. Thus, we found that some of the doses of melatonin (5 and 10 mg/kg) induce dendrite maturation of DCX-cells. This result is in the same line that our previous study in which we found that 8 mg/kg of melatonin favor dendrite complexity of DCX-cells in C57Bl6 mice [24]. Interestingly, the dose of 2.5 mg/kg of melatonin increased the number but not the complexity either decreased despair-like behavior. This result suggests that the dendrite maturation of newborn neurons is relevant for better performance in the FST, as was previously reported with antidepressants and environmental interventions [44,45]. However, this result did not discard that the prolong administration of 2.5 mg/kg of melatonin could revert depressive-like behavior and increase dendrite maturation. However, we consider that this hypothesis must be addressed in another study. In fact, in a current study of our group, we analyze hippocampal neurogenesis, microglial cells and the capability of melatonin to revert depressive-like behavior in male Balb/C mice expose to chronic mild stress. In this sense, other studies have reported the antidepressive-like effect of melatonin in rats exposed to chronic mild stress [20,46].

Even though several studies indicate the effects of melatonin on increasing DCX-cells and dendrite complexity [24,25,42,43,47], there are no data regarding the effects of melatonin on the distribution and dendrite complexity of DCX-cells in the dorsal- and ventral- DG. These aspects are relevant because an interesting regulation caused by other modulators of hippocampal neurogenesis with antidepressant-like effects, such as antidepressants (i.e., fluoxetine) or environmental interventions such as enrichment environment, have shown uniform results along the dorsal-ventral DG (for review see [41]). However, agomelatine that belongs to a novel class of antidepressants based on their action of melatonin and the antagonism of the serotonin receptor 5HT2c [48] has shown a specific effect on the maturation of adult newborn neurons only in the ventral-DG [49]. We here reported that the higher doses of melatonin (5 and 10 mg/kg) produce uniform effects in the dorsal- and ventral- DG in the increasing number and dendrite maturation of DCX-associated cells. Interestingly, a previous study did not find effects of melatonin on the absolute neurogenesis either on the distribution of newborn neurons in the dorsal-and ventral- DG [50]. However, it is important to consider the differences between both studies. We here used coronal slices for dorsal-DG and transversal slices for ventral-DG from Balb/C mice while in the other study authors used coronal sections from C3H/HeN mice [50]. As was previously stated, the method used in the present study allows us to achieve a more accurate measure of potential differences in dorsal versus ventral quantification [51]. However, the differences between the strains of mice used in both studies may be relevant [52]. Here, we used Balb/C mice which show medium to low levels of baseline adult hippocampal neurogenesis, but high relative numbers of surviving newborn cells in comparison to CD1 mice [52], and at least in one study was observed a very high sensitivity to activity-induced regulation [53]. However, C3H/HeN mice are melatonin-proficient [54] and it did not favor neurogenesis in both dorsal- and ventral- DG. However, melatonin just increased the effects of physical activity on neurogenesis in the dorsal-DG [50].

Moreover, melatonin may promote maturation of dendrite through microtubules reorganization. This process analyzed in vitro performed in non-neural precursor cells suggests the participation of protein kinase C (PKC) and Rho-associated kinase (ROCK) proteins [55,56,57]. In this sense, it is important to consider that DCX is a microtubule-associated protein. Also, melatonin modulates cytoskeleton- microtubule polymerization. Interestingly, the fact that a more significant proportion of DCX-cells show more complex dendrites supports the hypothesis that melatonin influences dendrite maturation which is necessary for the integration of new neurons in the hippocampal circuitry. However, the mechanism that underlay the effects of melatonin on dendrite maturation deserves exploring in an additional complex study, including in vitro precursor cells, which is currently conducting in our group. However, it is important to note that melatonin acts on four key events (proliferation, survival, dendrite maturation and axonal growth) of the adult hippocampal neurogenic process in Balb/C mice [25,26]. Also, melatonin could influence the dentate gyrus microenvironment by increasing the levels of neurotrophins or growth factors. In this regard, it has been shown that melatonin increases the levels of brain-derived neurotrophic factor in an in vitro cell culture model of neurons [58]. Interestingly, in our current conducting study, we observed that melatonin increases the secretion of the vascular endothelial growth factor from in vitro precursor cells and in the hippocampus of female Balb/C mice. Thus, these evidences support that the study of melatonin mechanisms to favor adult hippocampal neurogenesis is complex [25,26].

Also, our study showed the correlation between the dendrite maturation of DCX-associated cells with the decrease in despair behavior in mice treated with 10 mg/kg of melatonin. This fact is interesting because also 5 mg/kg of melatonin favored the increase number and maturation of DCX-cells; however, it has no significant correlation with the despair like behavior tested in the FST. Also, melatonin acts on other processes of neuroplasticity. For example, melatonin favors maturation of dendritic spines, axonal maturation and, the participation of the oligodendrocytes population [59,60]. Then, those antecedents support that melatonin complements its effects on neurogenesis with its antidepressant-like effect.

Nevertheless, this result strongly supports that melatonin modulates hippocampal neurogenesis and behavior in male Balb/C mice. Finally, our study provides support for melatonin as a critical modulatory factor that promotes plasticity in the DG and that its administration induces antidepressant-like effects. Thus, it may suggest that the study of the antidepressant-like effects of melatonin and its correlation with reversing altered behavior is worth pursuing.

## 4. Materials and Methods

### 4.1. Animals

Fifty-eight male Balb/C mice obtained from Harlan (Mexico city, Mexico). They were housed in standard laboratory cages under 12 h light/12 h dark cycles at a temperature of 23 ± 1 °C in the animal facilities of the National Institute of Psychiatry (city, state abbreviation if USA or Canada, country). The light/dark cycle corresponded to the timing of lights on (Zeitgeber time 0; ZT0) at 0700 hours and to the timing of lights off (Zeitgeber time 12; ZT12) at 1900 hours, respectively. The animals had access to food and water ad libitum. Mice were acclimatized to their new environment until the age of 8 weeks. All institutional and legal regulations regarding animal ethics and handling were followed for the in vivo experiments (IACUC SIC092025, 20 September 2011 by the Ethics committee of the National Institute of Psychiatry).

### 4.2. Melatonin Treatment

Melatonin was prepared fresh every day and dissolved in a minimum volume of pure ethanol plus saline solution (0.9% NaCl) to administrate 0.5, 1.0, 2.5, 5.0 and 10 mg/kg of body weight per mouse [34]. Tubes containing the melatonin solution were wrapped as we previously reported to prevent light-induced degradation [15]. The final volume of ethanol in the melatonin vehicle was less than 1%. Melatonin was administered once daily during 14 days at the beginning of the dark phase of the light-dark cycle via intraperitoneal (i.p.). The route of administration follows that absorption of melatonin occurs via any route, and it crosses all morpho-physiological barriers [61]. On day 16 of the protocol, mice were exposed to the Porsolt’s test to evaluate antidepressant-like behavior [15] (Figure 1).

### 4.3. Behavioral Testing in the Porsolt Forced Swim Test

The Porsolt swim test was used to evaluate the antidepressant-like action [62,63] of the chronic administration of MLT for 14 days. Thirty-six hours after the last injection, mice were habituated to the testing room for 30 min before the behavioral assay. Behavioral testing was performed at the end of the dark phase of the light/dark cycle (ZT23). The testing area was dimly lit to reduce stress or anxiety. Mice were gently placed in a cylinder (15 cm in diameter) filled to a depth of approximately 15 cm with water maintained at room temperature. The sessions were videotaped for the posterior analysis [58]. After testing, each mouse was gently dried, placed in a pre-heated holding cage with standard bedding, and covered by an absorbent paper towel for 30 minutes. Subsequently, mice were returned to their home cages [64]. The behavior of the animals was video recorded and analyzed blind to the experimental group with the ANY-maze behavioral tracking software (Stoelting Co., Wood Dale, IL, USA). The behavioral aspect detected was immobility detection (considering the minimal movements exerted by the animal to keep its head above water and floating). Thus, we analyzed the time spent immobile and number of episodes of immobility.

### 4.4. Tissue Processing for Immunohistochemistry

Two hours after the behavioral test, mice were killed by an overdose of ketamine and perfused with a 4% paraformaldehyde solution. Brains were dissected from the skull and kept in 4% paraformaldehyde for seven days. Brains were cryoprotected by immersing them in a 30% sucrose solution and left there until tissues sank to the bottom. Next, brains were cut into 40 micrometers sections with a sliding microtome (Leica, Buffalo Grove, IL, USA). The sections stored at 4 °C in a cryoprotectant solution (25% ethylene glycol and 25% glycerin in 0.05 M phosphate buffer). Brains were sectioned following a previous report to obtain representative sections of the ventral and dorsal dentate gyrus [65]. This method allows us to achieve a more accurate measure of potential differences in dorsal versus ventral quantification. Stainings were done for every twelve-section covering the dorsal or ventral DG. Sections were stained following the free-floating immunohistochemistry method and pretreated for DCX by incubation in citrate buffer (pH 6.0) for 30 min at 95 °C followed by three washes in cold citrate buffer (pH 6.0) for 10 minutes each [15].

### 4.5. Quantification of Doublecortin-Labelled Cells

Positive cells for the DCX marker were identified with a specific antibody to DCX (Santa Cruz Biotech, Santa Cruz, CA, USA) and visualized with the peroxidase method [24,65]. Cells were counted exhaustively using a 40× objective. Counting was done as previously described using the modified optical dissector method under bright-light microscopy (DM500 microscope equipped with a video camera ICC50; Leica, Buffalo Grove, IL, USA). The cells appearing in the uppermost focal plane were excluded to avoid over-sampling [59]. The resulting numbers were multiplied by six to obtain the estimated total number of DCX-associated cells per granule cell layer.

### 4.6. Categorization of Dendritic Morphology of DCX-Positive Cells

DCX-labelled cells were classified according to their morphological appearance, as described previously [26]. Six categories established on the dendrite morphology: (a) DCX-labelled cells without dendrites; (b) cells showing shorter dendrites than the soma size; (c) cells with dendrites slightly larger than the soma size; (d) cells with dendrites size longer than category “c”; (e) cells with more mature appearance showing one primary dendrite with one branching point (node); (f) cells with mature appearance showing more than 3 nodes and reaching the molecular layer. Absolute quantification was performed by calculating the percentage of cells in each category and the total number of DCX-positive cells.

### 4.7. Statistical Analysis

The analysis was performed using SigmaPlot 12.0 software (SystatSoftware Incorporation, San Jose, CA, USA). We present the results are presented as the mean ± standard error of the mean (SEM). Some results were analyzed with a one-way ANOVA followed by the Bonferroni post hoc test. However, when the normality test failed, we applied a non-parametric Kruskal-Wallis one-way ANOVA on ranks followed by the multiple comparison procedures within the Dunn’s method. Comparison between melatonin doses (factor A) and category or region of the dentate gyrus (dorsal or ventral; factor B) were analyzed with a two-way ANOVA followed by the Bonferroni post hoc test. Also, the correlation between two variables was analyzed with the Pearson correlation. Differences were considered statistically significant at *p* ≤ 0.05.

## Abbreviations

DG	Dentate gyrus
DCX	Doublecortin
FST	Forced-swim test
PKC	Protein kinase C
ROCK	Rho-associated kinase
